# A Novel Approach for Improving Gait Speed Estimation Using a Single Inertial Measurement Unit Embedded in a Smartphone: Validity and Reliability Study

**DOI:** 10.2196/52166

**Published:** 2024-08-13

**Authors:** Pei-An Lee, Wanting Yu, Junhong Zhou, Timothy Tsai, Brad Manor, On-Yee Lo

**Affiliations:** 1 Hinda and Arthur Marcus Institute for Aging Research, Hebrew SeniorLife, Boston, MA, United States; 2 Division of Gerontology, Beth Israel Deaconess Medical Center, Boston, MA, United States; 3 Harvard Medical School, Boston, MA, United States

**Keywords:** smartphone app, gait speed, dual-task walking, validity, reliability, mobile phone

## Abstract

**Background:**

Gait speed is a valuable biomarker for mobility and overall health assessment. Existing methods to measure gait speed require expensive equipment or personnel assistance, limiting their use in unsupervised, daily-life conditions. The availability of smartphones equipped with a single inertial measurement unit (IMU) presents a viable and convenient method for measuring gait speed outside of laboratory and clinical settings. Previous works have used the inverted pendulum model to estimate gait speed using a non–smartphone-based IMU attached to the trunk. However, it is unclear whether and how this approach can estimate gait speed using the IMU embedded in a smartphone while being carried in a pants pocket during walking, especially under various walking conditions.

**Objective:**

This study aimed to validate and test the reliability of a smartphone IMU–based gait speed measurement placed in the user’s front pants pocket in both healthy young and older adults while walking quietly (ie, normal walking) and walking while conducting a cognitive task (ie, dual-task walking).

**Methods:**

A custom-developed smartphone application (app) was used to record gait data from 12 young adults and 12 older adults during normal and dual-task walking. The validity and reliability of gait speed and step length estimations from the smartphone were compared with the gold standard GAITRite mat. A coefficient-based adjustment based upon a coefficient relative to the original estimation of step length was applied to improve the accuracy of gait speed estimation. The magnitude of error (ie, bias and limits of agreement) between the gait data from the smartphone and the GAITRite mat was calculated for each stride. The Passing-Bablok orthogonal regression model was used to provide agreement (ie, slopes and intercepts) between the smartphone and the GAITRite mat.

**Results:**

The gait speed measured by the smartphone was valid when compared to the GAITRite mat. The original limits of agreement were 0.50 m/s (an ideal value of 0 m/s), and the orthogonal regression analysis indicated a slope of 1.68 (an ideal value of 1) and an intercept of −0.70 (an ideal value of 0). After adjustment, the accuracy of the smartphone-derived gait speed estimation improved, with limits of agreement reduced to 0.34 m/s. The adjusted slope improved to 1.00, with an intercept of 0.03. The test-retest reliability of smartphone-derived gait speed was good to excellent within supervised laboratory settings and unsupervised home conditions. The adjustment coefficients were applicable to a wide range of step lengths and gait speeds.

**Conclusions:**

The inverted pendulum approach is a valid and reliable method for estimating gait speed from a smartphone IMU placed in the pockets of younger and older adults. Adjusting step length by a coefficient derived from the original estimation of step length successfully removed bias and improved the accuracy of gait speed estimation. This novel method has potential applications in various settings and populations, though fine-tuning may be necessary for specific data sets.

## Introduction

Gait speed, often considered the sixth vital sign, is a meaningful health indicator that is routinely assessed within clinical and research settings [1-4]. As a biomarker, gait speed is an integrated and reproducible measure of mobility that helps to identify the risk of falls, disability, hospitalization, and even mortality [5,6]. It is also responsive to intervention and is often used to evaluate the effectiveness and progression of rehabilitation programming [3,7]. Within clinical settings, gait speed is most commonly measured by using a stopwatch to record the time taken to walk a short distance [8]. This approach, while validated, requires another person’s assistance and is often inaccurate due to human error or bias. Within the laboratory, gait speed is typically measured using motion capture systems or instrumented walkways. This approach increases measurement accuracy yet requires trained personnel and expensive equipment, limiting its accessibility and feasibility for frequent, longitudinal monitoring. Moreover, neither of these clinical or laboratory-based approaches is well-suited for monitoring changes over time via higher-frequency assessments, and neither approach affords the assessment of gait speed assessment under unsupervised conditions during daily life activities. There is thus a need to develop new methods for the low-cost, easy, and accurate measurement of gait speed for use within both clinical and remote, unsupervised settings.

Smartphones, now widely used by younger and older adults and equipped with a high-quality inertial measurement unit (IMU), present a viable and convenient opportunity to objectively measure gait speed outside of laboratory and clinical settings. Previous studies have demonstrated that gait speed can be accurately estimated using multiple IMUs attached to various parts of the body (eg, the trunk and ankles) [9-11]. However, this approach requires specialized equipment that may be difficult to don and doff. Alternatively, studies have attempted to estimate gait speed using a single, research-grade IMU attached to the trunk of the body. In this case, the inverted pendulum model was used to determine gait speed by estimating stride distance and dividing it by stride time, determined by acceleration peaks induced by consecutive heel strikes [12-15]. This approach, while promising, still requires trained personnel for equipment setup and may introduce bias in gait speed estimation, especially at a faster or slower gait speed [13-15]. In this study, we examined the validity and reliability of deriving gait speed from a smartphone IMU with the phone being carried in the individual’s front pants pocket when walking.

This study aimed to validate and test the reliability of a smartphone IMU–based gait speed measurement placed in the user’s pants pocket in both healthy young and older adults during normal and dual-task walking. The validity of the smartphone-based approach was determined by comparing the estimated step length and gait speed to measurements taken by gold standard instrumentation. Based on initial results, we further examined the use of improving gait speed estimations using a coefficient relative to the estimation of step length as determined by the smartphone. The test-retest reliability of adjusted and unadjusted estimations was examined within both laboratory and unsupervised, real-life home settings. This study is expected to provide valuable insights applicable to a wide range of clinical and everyday gait monitoring scenarios.

## Methods

### Study Participants

We conducted a design control verification and validation study enrolling younger (aged 20‐50 years) and older (aged 65‐90 years) adults between 2016 and 2018. As part of our comprehensive assessment at baseline, we measured key demographic characteristics of the participants, including sex, age (in years), height (in meters), body weight (in kilograms), and ethnicity. We included those who had active Wi-Fi service in their homes and who were able to use the smartphone app by themselves after training. Assistive devices were allowed if participants normally used them when walking. Individuals were excluded if they were hospitalized within the last 6 months; were unable to walk without assistance; self-reported major neuromuscular, cardiovascular, or metabolic disease; had lower-extremity ulcers or amputations; or self-reported pain significantly affecting their gait.

### Ethics Approval

This study was approved by the Hebrew SeniorLife Institutional Review Board (approval IRB-2015‐40).

### Smartphone App

Our team previously created an iOS smartphone–based application (app) that uses the phone’s IMU sensor to record movements while walking freely at a self-selected speed (ie, normal walking condition) and while walking and concurrently performing a serial-subtraction task (ie, dual-task walking condition) with the phone placed in the user’s pants pocket [16]. The app was designed to recreate a commonly used dual-task gait assessment that is typically performed in a laboratory setting. The initial development process involved a detailed analysis of requirements, followed by iterative design and user feedback–driven enhancements, ensuring both user-friendliness and adherence to clinical standards. The app includes a series of instructions for the participants to help ensure assessment reliability. Once the participant presses the “Start” button and places the phone in their preferred front pocket, the app provides auditory instructions for each walking trial via the iPhone speaker, including a randomly generated starting number for the serial-subtraction task for dual-task trials, and cues for the start and end of the trial. These cues trigger the acquisition of 3-axis accelerometer, gyroscope, and magnetometer data at a 100-Hz sampling rate. The data sets are saved on the phone’s internal storage and automatically transmitted via Wi-Fi to a remote, cloud-based data server for offline analysis. The validity and reliability of the smartphone app, especially in temporal information (ie, stride time), have been established [16-18]. Additionally, its applicability extends to various populations, including individuals with Parkinson disease and older adults diagnosed with blood cancer [18-21].

### Study Procedures

#### Overview

Each participant completed 2 laboratory visits separated by at least 1 week. Between these visits, participants were asked to complete the walking assessment using the app at home once a day on 3 separate days. Participants were instructed to wear comfortable pants or shorts with front pockets for each visit. Our previous work indicated that stride time estimations were unaffected by pocket tightness [16].

#### Laboratory Assessments

The same procedures were used on both laboratory visits. Within each visit, participants completed the walking assessment using the app 3 times, that is, 3 pairs of normal and dual-task walking trials. For each walking trial, participants walked around an oval-shaped, 24-m indoor track. The GAITRite mat (CIR Systems, Inc) was placed along one long side of the track. Each trial began with participants standing just behind the beginning of the mat to ensure that the first footfall of each trial was captured by the mat. Participants were instructed to use the app to start and finish each trial. Stride time, step length, and gait speed obtained from the first pass over the GAITRite mat were considered the gold standard and aligned the data sets to enable direct comparison of GAITRite and smartphone app data for each identified step.

#### Home Assessments

At home, participants were instructed to use the app to complete 1 normal walking and 1 dual-task walking on 3 separate days in between the 2 laboratory visits. The app provided the same instructions as given during the laboratory visit. The participants were instructed to walk continuously along the longest hallway or unobstructed path in their home, making 180-degree turns at each end, throughout the trial. We believe this setup can be effective in remote settings, provided that a flat and consistent walking surface is available, external interference is minimal, and stable Wi-Fi is present.

### Data Analysis

#### Gait Speed Estimation

All data analyses, graphics generation, and statistical analyses were performed using programs developed in-house within MATLAB (R2022b, MathWorks) and SPSS (version 20; SPSS Inc).

For the data obtained from the smartphone, the raw time series 3-axis accelerometer and gyroscope data were transformed from the smartphone local coordinate system to an earth global coordinate system using a quaternion rotation matrix. This transformation ensured that the z-axis of the data aligned with the vertical line of gravity. After the transformation, each z-axis time series was filtered using a Butterworth filter [16,22], chosen for its smooth frequency response and minimal signal distortion, which worked well in our previous published study [16]. The time series data obtained from the accelerometer and gyroscope sensors contained alternating peaks of high and low amplitude that corresponded to the heel-strike and toe-off events [16]. Stride time was defined as the time between 2 consecutive heel strikes of the same foot, which was calculated by determining the number of data points between 2 heel strikes and dividing by 100-Hz sampling frequency. The step length was estimated using a simple inverted pendulum model, which uses the participant’s leg length (*l*) and the change in height of the smartphone’s vertical position over each step (*h*) as the following equation:


(1) $$Step\;length=2*\sqrt{2*h*l-h^{2}}$$

In particular, the vertical position was derived by doubly integrating the vertical acceleration data (z-axis) and high-pass filtering the outcome using a fourth-order zero-lag Butterworth filter with a 0.11-Hz cutoff frequency to remove any integration drift [12,23]. The fourth order of the filter ensures effective attenuation of frequencies below 0.11 Hz, substantially reducing low-frequency drift caused by noise in the acceleration data. Additionally, the zero-lag design of the Butterworth filter preserves the temporal accuracy of the signal, ensuring precise alignment of the filtered output with the real-time events being measured. Gait speed for each stride was then calculated by dividing stride length (ie, step length multiplied by 2) by the corresponding stride time. Gait variables thus included step length and gait speed from both GAITRite mat and smartphone app data were used for the statistical analyses.

#### The Step Length Adjustment Process

**Figure 1.**
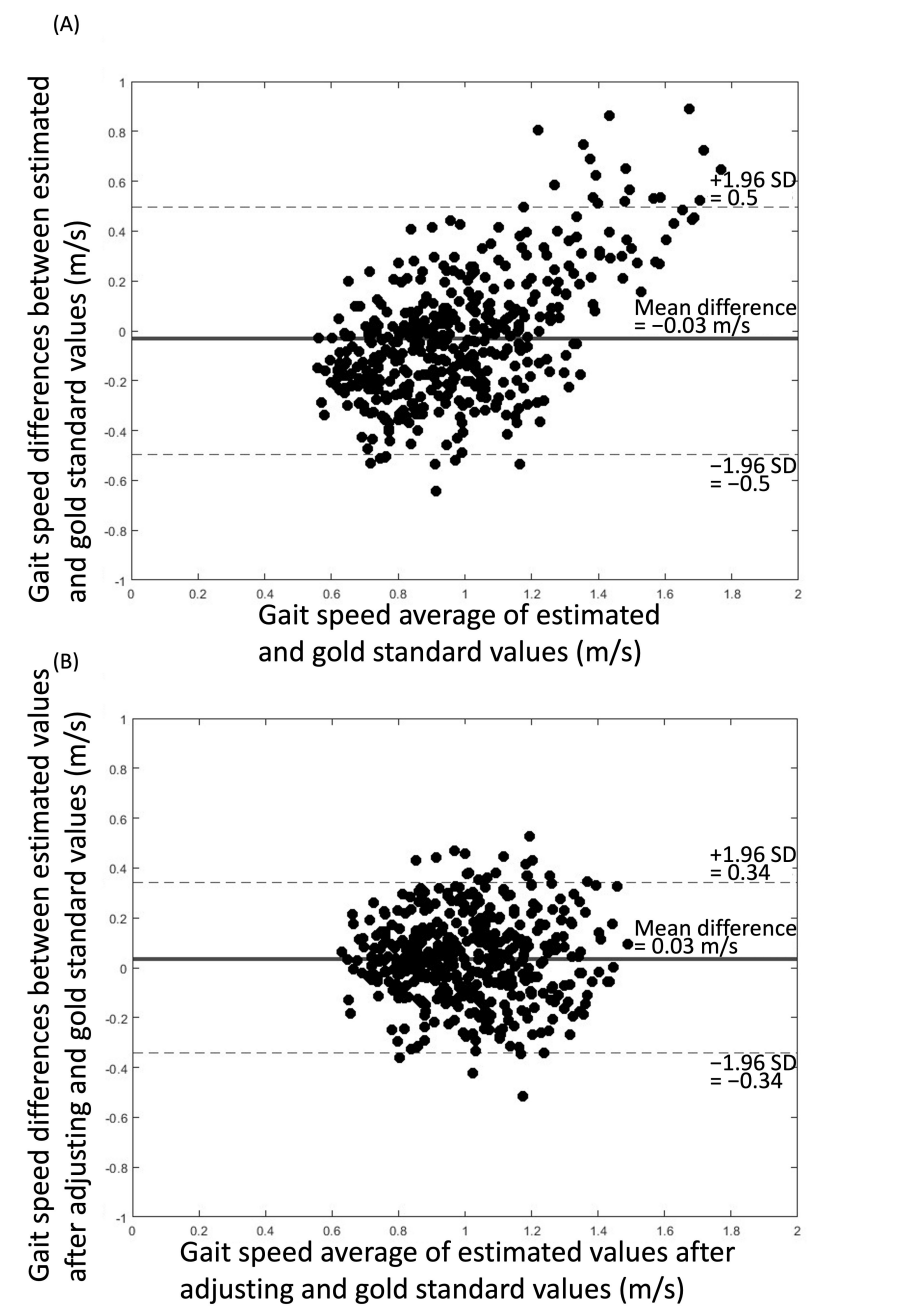
Bland-Altman plot for gait speed when using the smartphone app–based assessment compared to GAITRite mat before (A) and after (B) adjusting by coefficients relative to the original estimation of step length. The solid lines are the average difference, and the dashed lines are the limit of agreements (SD 1.96).

**Figure 2.**
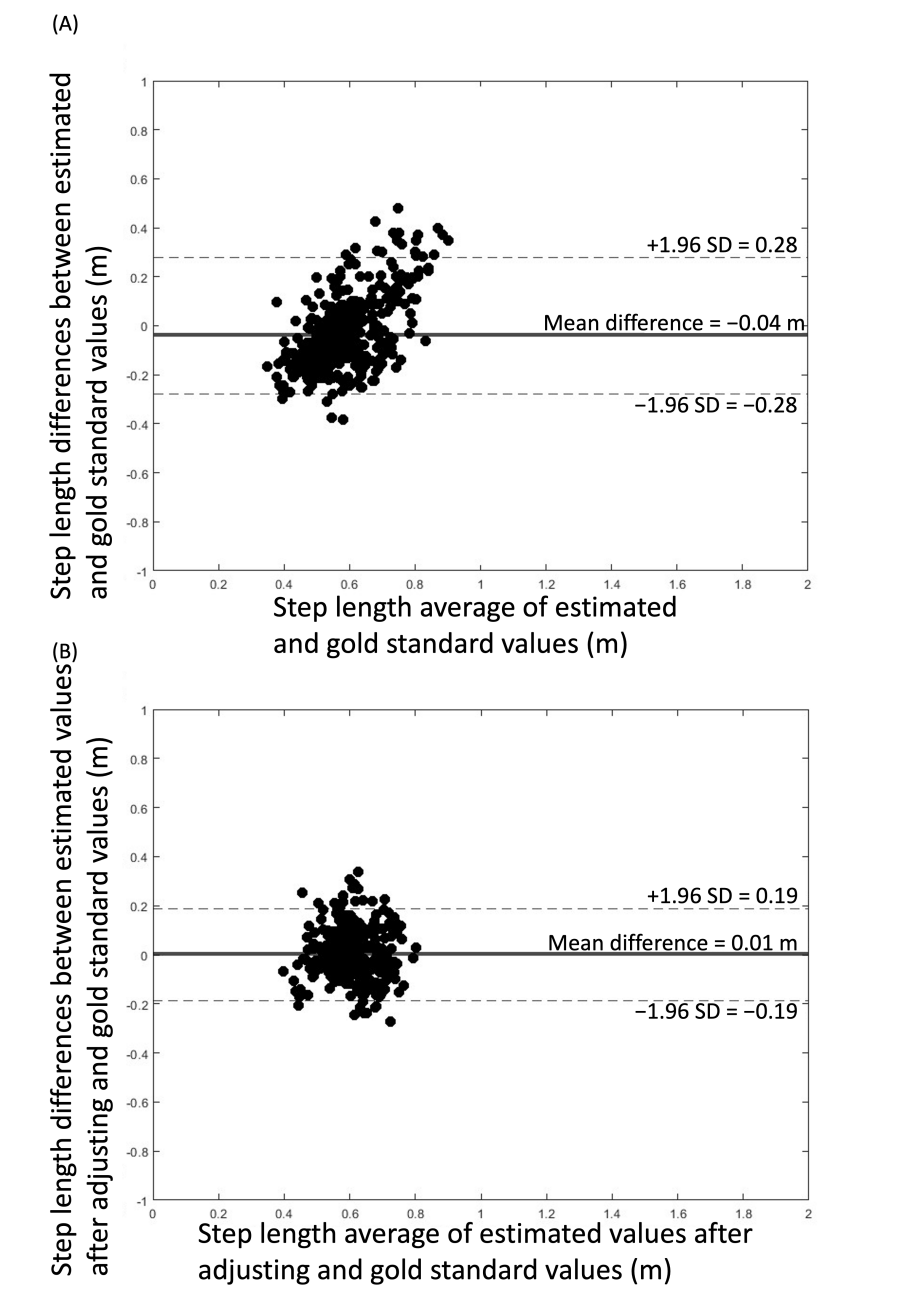
Bland-Altman plot for step length when using the smartphone app–based assessment compared to GAITRite mat before (A) and after (B) adjusting by coefficients relative to the original estimation of step length. The solid lines are the average difference, and the dashed lines are the limit of agreements (SD 1.96).

After calculating the validity of the gait speed and step length derived from the smartphone app and GAITRite (see Statistical Analysis section), we observed a bias between smartphone app and GAITRite measurements for both gait speed and step length across all participants, walking conditions, and laboratory trials. That is, as the step length (average of the smartphone app and GAITRite mat measurement) increased, the step length derived from the app became increasingly greater than the corresponding values derived from the GAITRite mat. This trend was independent of the walking condition and study visit order (Figures 1A and 2A). To address this, we systematically tested various intervals, starting from 0.1, 0.15, 0.2 m, and all the way up to 0.5 m, with a 0.05-m increment, for adjusting the step length. Our analysis revealed that an interval of 0.3 m consistently produced the best validity, evidenced by a slope of 1 and an intercept close to 0 m in the relationship between our adjusting measurements and those from the GAITRite mat. These findings indicate a robust linear correlation and minimal systematic bias. This method was designed for its ability to adjust the step length estimation, aiming to align more closely with the gold standard GAITRite measurements, particularly addressing the identified bias across different step lengths. The intervals were chosen based on a detailed stepwise iterative approach, where each interval was evaluated for accuracy and precision. This approach allowed us to capture the nonlinear relationship between the app-derived and GAITRite-derived step lengths across the range of observed values (Table 1).


(2) Adjustedsteplength=coefficients∗steplength

**Table 1.** Coefficients that were used to adjust step length and gait speed.

		Step length 0.2‐0.5 m	Step length 0.5‐0.8 m	Step length 0.8‐1.1 m
**Coefficients**				
	All data points	1.37	1.02	0.74
	Only normal walking data points	1.40	1.04	0.74
	Only dual-task walking data points	1.36	1.01	0.73

#### Statistical Analysis

The validity of app-derived gait speed and step length was examined by estimating their agreement with corresponding gait data derived from the gold standard GAITRite mat using a Passing-Bablok orthogonal regression model, which is suitable for comparing different measurement methods while acknowledging measurement error [24]. This statistical method was chosen for its robustness in comparing methods with measurement error, providing a more accurate assessment of agreement between different measurement systems. Initial sources and seminal works on the Passing-Bablok regression model highlight its suitability for medical and biomechanical research applications [24]. The magnitude of error between the gait data from the app and the GAITRite mat was calculated for each stride, and a Bland-Altman plot was produced to visualize this error as a function of gait data, which was the average of the gait speed calculated by the app and the GAITRite mat. Test-retest reliability was assessed for all derived gait parameters from both the smartphone app and GAITRite mat using several intraclass correlation coefficients (ICCs). For each of the following two conditions, we computed ICC separately for normal and dual-task walking trials: (1) across trials within each laboratory assessment, and (2) across trials over the 3 home assessments. The unit of interest was the average gait speed in strides derived from each trial of the same condition (ie, normal or dual-task walking; ICC (1, 1)). We considered ICC values greater than 0.75 as excellent reliability, 0.6‐0.75 as good reliability, 0.4‐0.6 as fair reliability, and less than 0.4 as poor reliability [25].

## Results

### Overview

In total, 12 healthy young adults (6 female adults and 6 male adults; age: mean 29.1, SD 4.4 years; height: mean 168.7, SD 13.1 cm; and body mass: mean 74.0, SD 13.9 kg) and 12 healthy older adults (8 female adults and 4 male adults; age: mean 72.0, SD 6.4 years; height: mean 165.6, SD 9.0 cm; and body mass: mean 71.6, SD 16.3 kg) participated in the study.

A total of 24 participants completed 96 passes over the GAITRite mat with the phone placed in their pocket during the laboratory visits. Each session comprised 2 normal walking trials and 2 dual-task walking trials, yielding 4‐5 strides per trial for each participant. This resulted in a data set comprising a total of 442 strides for direct comparison of gait speed, step length, and stride time estimations between the GAITRite mat and smartphone app approaches. Average normal and dual-task gait speeds for the younger and older groups are presented in Table 2.

**Table 2.** Means (SDs) and minimum (min)-maximum (max) of the spatiotemporal parameters during normal and dual-task walking in the younger and older groups.

Tests		Normal walking						Dual-task walking					
		Gait speed (m/s)		Step length (m)		Stride time (s)		Gait speed (m/s)		Step length (m)		Stride time (s)	
		Mean (SD)	Min-max	Mean (SD)	Min-max	Mean (SD)	Min-max	Mean (SD)	Min-max	Mean (SD)	Min-max	Mean (SD)	Min-max
**GAITRite**													
	Younger	1.16 (0.08)	1.06‐1.35	0.65 (0.04)	0.57‐0.70	1.15 (0.08)	1.01‐1.24	1.02 (0.09)	0.94‐1.23	0.62 (0.03)	0.56‐0.66	1.23 (0.12)	0.96‐1.34
	Older	1.00 (0.06)	0.94‐1.18	0.57 (0.02)	0.54‐0.62	1.18 (0.05)	1.04‐1.22	0.95 (0.07)	0.81‐1.12	0.56 (0.02)	0.50‐0.60	1.22 (0.05)	1.07‐1.27
**Smartphone app: before adjusting**													
	Younger	1.11 (0.25)	0.90‐1.77	0.61 (0.11)	0.53‐0.92	1.12 (0.10)	0.93‐1.22	0.96 (0.24)	0.82‐1.66	0.57 (0.12)	0.47‐0.93	1.22 (0.14)	0.91‐1.34
	Older	0.97 (0.18)	0.58‐1.24	0.54 (0.10)	0.33‐0.66	1.15 (0.04)	1.06‐1.20	0.90 (0.18)	0.54‐1.18	0.52 (0.09)	0.34‐0.63	1.18 (0.05)	1.08‐1.27
**Smartphone app: after adjusting**													
	Younger	1.13 (0.12)	1.01‐1.36	0.63 (0.02)	0.60‐0.67	—^a^	—	1.01 (0.14)	0.91‐1.25	0.60 (0.03)	0.55‐0.69	—	—
	Older	1.04 (0.11)	0.80‐1.27	0.59 (0.06)	0.45‐0.67	—	—	1.01 (0.11)	0.75‐1.20	0.59 (0.05)	0.47‐0.65	—	—

a Not applicable.

### Validity and Reliability of the Original Estimated Gait Speed and Step Length

**Figure 3.**
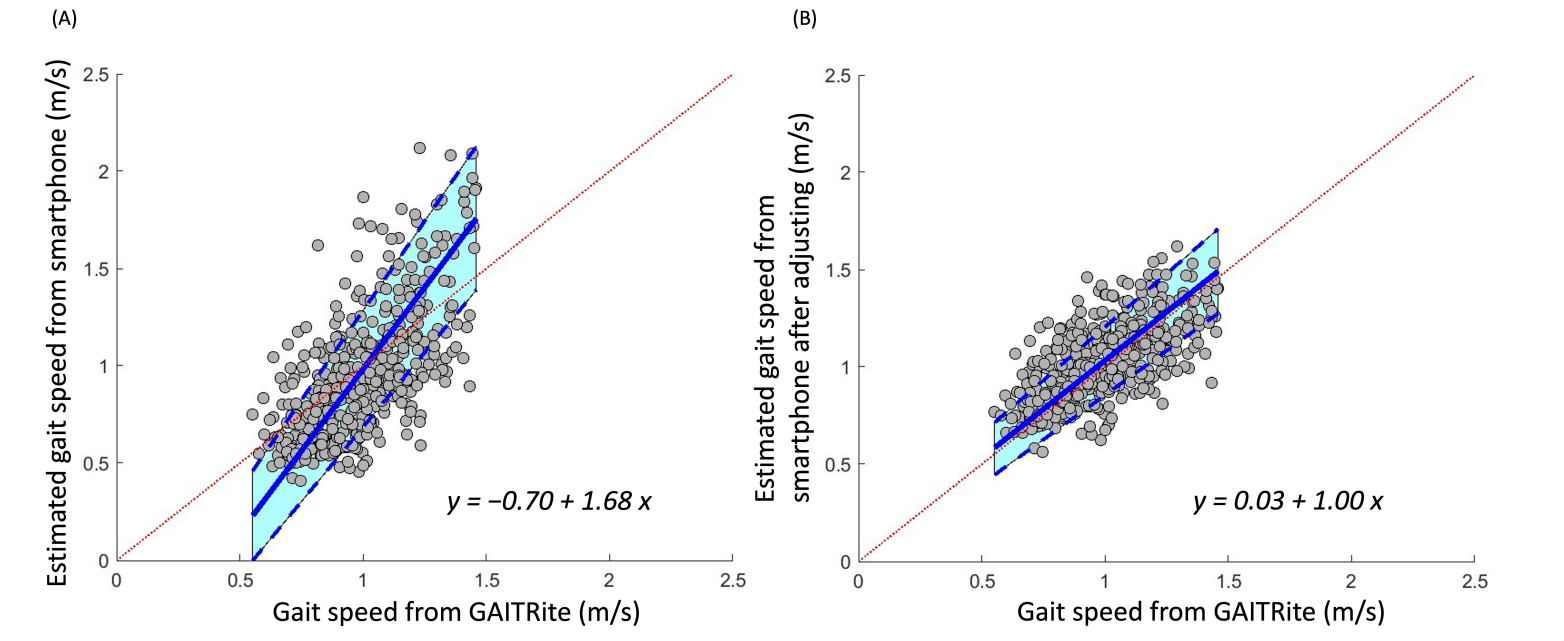
Relationship between gait speed as derived from a smartphone app–based assessment and from a GAITRite mat before (A) and after (B) adjusting by coefficients relative to the original estimation of step length. The Passing-Bablok orthogonal best-fit line of these data had a slope of approximately 1 and an intercept of 0 after adjusting.

**Figure 4.**
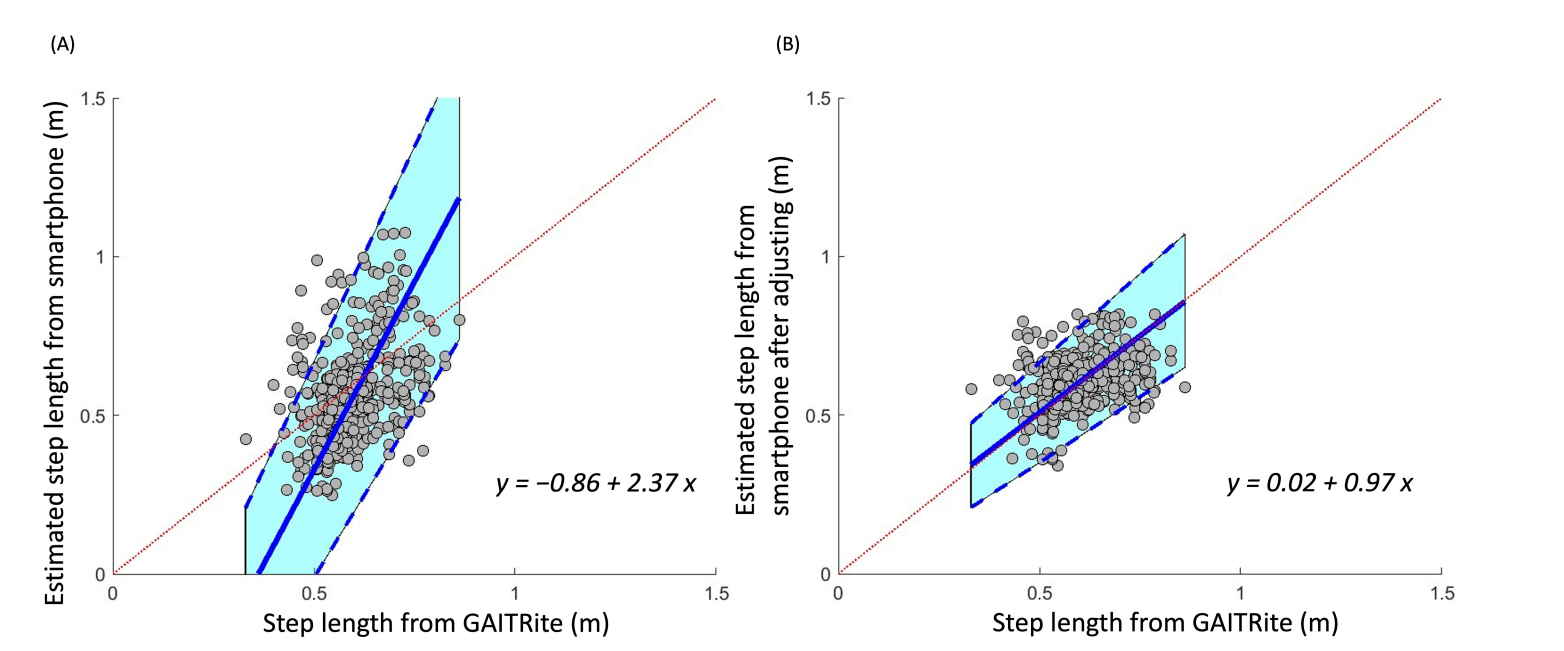
Relationship between step length as derived from a smartphone app–based assessment and from a GAITRite mat before (A) and after (B) adjusting by coefficients relative to the original estimation of step length. The Passing-Bablok orthogonal best-fit line of these data had a slope of approximately 1 and an intercept of 0 after adjusting.

Average normal and dual-task gait speeds within each group, as estimated from the smartphone app, were largely similar to those derived from the GAITRite mat (Table 2). Smartphone-derived gait speed and step length demonstrated good validity as compared with the GAITRite mat. The close-to-zero average magnitude of biases for gait speed and step length was −0.03 m/s and −0.04 m, and the limits of agreement were 0.5 m/s and 0.28 m, respectively (Figures 1A and 2A). Orthogonal regression analysis revealed that the slope and intercept of the gait speed and step length derived from the app were 1.68 (intercept=−0.70) and 2.37 (intercept=−0.86), respectively (Figures 3A and 4A).

Good to excellent test-retest reliabilities (0.64‐0.86) were demonstrated for the average app-derived gait speed across trials within each laboratory assessment, as well as over the 3 home assessments, for both normal and dual-task walking in young and older adults (Table 3).

**Table 3.** Test-retest reliability of the laboratory- and home-based assessments of average gait speed in strides.

Tests				Normal walking			Dual-task walking		
				ICC^a^	*P* value	95% CI	ICC	*P* value	95% CI
**GAITRite mat**									
	Laboratory assessment within visit			0.94	<.001	0.88-0.97	0.90	<.001	0.81-0.95
**Before adjusting: smartphone app**									
	Laboratory assessment within visit			0.86	<.001	0.73-0.93	0.77	<.001	0.58-0.88
	Home assessment			0.64	<.001	0.41-0.81	0.71	<.001	0.49-0.86
**After adjusting: smartphone app**									
	Laboratory assessment within visit			0.72	<.001	0.52-0.85	0.75	<.001	0.56-0.87
	Home assessment			0.62	<.001	0.38-0.80	0.76	<.001	0.57-0.89

a ICC: intraclass correlation coefficient.

### Validity and Reliability of Smartphone-Derived Gait Speed After Adjusting Step Length by a Coefficient Relative to the Original Estimation of Step Length

After iteratively calculating the validities and reliabilities of the different step length zone thresholds as intervals, results indicated that 0.3 as an interval appeared to produce the best validity (Table 4). After adjusting step length with this coefficient relative to the original estimation of step length, the average gait speed in the younger group during normal and dual-task walking was 1.13 (SD 0.12) and 1.01 (SD 0.14) m/s, respectively; and the average gait speed in older group during the 2 conditions was 1.04 (SD 0.11) and 1.01 (SD 0.11) m/s, respectively (Table 2). These adjusted estimations of gait speed and step length derived from the smartphone app demonstrated a considerably smaller bias trend compared to the original estimated data (Figures 1 and 2). For gait speed and step length, mean differences were 0.03 m/s and 0.01 m, and limits of agreement were 0.34 m/s and 0.19 m, respectively (Figures 1B and 2B). Orthogonal regression analysis revealed that the validity of gait speed and step length derived from the app were higher with those measured by the GAITRite mat after adjusting, as indicated by one and close-to-one slopes, which were 1.00 (intercept=0.03) and 0.97 (intercept=0.02), respectively (Figures 3B and 4B).

**Table 4.** The slope and intercept for the Passing-Bablok orthogonal best-fit line after adjusting using stepwise iterative approach to define different step length zones by dividing the original estimation of step length using multiple zone thresholds as intervals from 0.1 to 0.5.

Intervals	Slope	Intercept
0.10	0.97	0.23
0.15	0.86	0.16
0.20	0.91	0.12
0.25	1.00	0.04
0.30	1.00	0.03
0.35	1.13	−0.09
0.40	1.23	−0.18
0.45	1.35	−0.28
0.50	1.40	−0.34

In general, the test-retest reliability of adjusted estimations of gait speed was good to excellent (0.62‐0.76) between laboratory assessments, and between home assessments, for both normal and dual-task walking in the younger and older groups (Table 3).

## Discussion

This study indicated that using a smartphone app placed in the front pocket of an individual’s pants or shorts can produce valid estimates of gait speed under both normal and dual-task walking conditions in healthy younger and older adults. Moreover, we demonstrated that the accuracy of gait speed estimation using the inverted pendulum model approach can be improved by adjusting the estimated values with coefficients determined by the gold standard measurement of step length. This improved smartphone app–based approach provided high test-retest reliability within a supervised laboratory environment and within the unsupervised home setting.

The current results revealed that the pendulum model approach to calculating gait speed from a single IMU placed in the pocket overestimated step length and therefore gait speed if the step length derived from the GAITRite mat was greater than 0.8 m, yet underestimated both values if the step length was less than 0.5 m. Through visual inspection of published work, it appears that this observed bias in step length estimation is common, even when the IMU is secured to the participant’s trunk [13,15]. To our knowledge, however, no one has reported this observation, and no adjustment has been made to correct the bias.

Our work suggests that a simple adjustment of step length for those steps that are relatively short, or relatively long, removes the observed bias and improves the accuracy of gait speed estimation. The current results suggested that the original estimated step length from the smartphone app between 0.5 and 0.8 m may not need to be adjusted as its coefficient was close to 1. Comparing our smartphone app with gold standard measurements, it is evident that in scenarios involving normal or dual-task walking, the app maintains robust performance with small bias across younger and older groups, different walking conditions, and different testing settings (laboratory and home). This implies that the described method can be used accurately within numerous applications. The simple inverted pendulum model requires only 2 pieces of information, namely, the participant’s leg length and the change in height of the smartphone’s vertical position during each step (from heel strike to contralateral heel strike). We suspect that several factors may contribute to the observed bias. There may be nonlinear relationships between step length and the combinations of changes in the height of the smartphone’s vertical position and leg length. This could indicate inherent limitations in the inverted pendulum model. Exploring these factors further may thus offer insights into optimizing the model for different IMU locations and gait patterns, enhancing the applicability of our method across diverse populations and conditions.

The results from this study not only reinforce the reliability of the approach to estimate gait speed under different walking conditions in ambulatory older adults without overt disease but also suggest its applicability to all IMU data. Good to excellent test-retest reliability of results was observed across younger and older groups, different walking conditions (normal walking and dual-task walking), and different testing settings (laboratory and home). This implies that the described method can be used effectively within numerous applications. Moreover, the test-retest reliability observed in this study was noticeably higher than a previous report that used the pendulum model without adjustment [15]. Incorporating a wide range of ages from young to old in this study allowed us to encompass a broader spectrum of gait speeds and step lengths, which enhanced the generalizability of our findings. The observed consistency in the adjustment coefficients across these age groups suggests that the pendulum model approach we used is robust and applicable across a diverse age demographic. This inclusivity in our research approach enriches its relevance and widens its potential impact, thus making our findings more applicable in real-world scenarios where age-related variations in gait are common.

The coefficients established from our cohort may be applicable to other data sets where the populations and the settings of the devices (smartphone), including sampling rate, are similar; yet, the coefficients may need to be fine-tuned for different populations and devices, especially when a gold standard measurement is available. Our coefficients performed well in our data set, which involved a wide range of step length and gait speed in both healthy young and older populations. In terms of future application to other data sets, this method holds significant potential for various areas. For data sets where a gold standard method is unavailable, our coefficients are worth trying to adjust the original estimated value. If the population (ie, healthy young and healthy older adults) and tool (ie, a smartphone app in the pants pocket) are similar to that tested and used in this study, implementing this type of adjustment is expected to improve the accuracy and reliability of the estimates. For data sets where a gold standard is available, researchers are recommended to develop their own coefficients based on the unique characteristics of each data set, thereby improving the method’s precision and applicability to specific contexts.

Further research is required to determine the generalizability of our method to broader populations, especially in cases where a gold standard method is not available. The original step length estimations in this study were classified into 3 categories, which may decrease the test-retest reliability of those step length data close to the margin of categories. To address this, larger data sets are needed to establish a continuous adjustment approach without the need to classify the data into categories. Additionally, refining the inverted pendulum model to better accommodate the nonlinear relationship between step length and smartphone vertical displacement could enhance accuracy. The applicability of the method to individuals with abnormal gait patterns should also be explored. Despite these limitations, this study provided evidence that gait speed can be accurately and reliably estimated with minimum information (leg length) in young and older adults during normal and dual-task walking based upon a single IMU-embedded smartphone placed in the pocket.

## Abbreviations

ICC: intraclass correlation coefficient
IMU: inertial measurement unit
